# The Performance of Microfiltration Process for Purifying Lactic Acid in the Fermented Broth of Kitchen Waste

**DOI:** 10.3390/membranes13030280

**Published:** 2023-02-27

**Authors:** Yan Guo, Chenglong Li, Hongjun Zhao, Ming Gao, Qunhui Wang

**Affiliations:** 1 Department of Environmental Engineering, University of Science and Technology Beijing, 30 Xueyuan Road, Haidian District, Beijing 100083, China; 2 Department of Environmental Engineering, Tianjin College, University of Science and Technology Beijing, Tianjin 301830, China

**Keywords:** kitchen waste, fermentation broth, lactic acid, microfiltration, membrane, flushing

## Abstract

Fermentation broth is plentiful with lactic acid, an important chemical applied in many fields, such as food processing, the chemical industry, and cosmetics. However, the purification of the lactic acid from the broth is still troublesome, when considering the economy. This study first investigated the purification performance of microfiltration (MF) membrane technology for a fermentation broth from kitchen waste. The effect of operation pressure, broth pH, and membrane flushing mode on the membrane filtration performance were investigated. In addition, the change in filtration performance over the increase in cycle time was also investigated. The results showed that under the optimum pressure of 100 KPa, pH of 6.0, and a backflushing mode with deionized water for 3 min, the best performance was achieved, with chroma removal, turbidity removal, protein removal and total sugar removal efficiencies of 60, 92.8, 57.64 and 32.93%, respectively. The results indicated that the MF process could be a desirable broth purification process to some extent, and it is promising in actual application. The MF process combined with other post-purification processes will form the ideal process system, which should be explored in future research.

## 1. Introduction

Lactic acid (LA) is an important chemical, which exists widely in nature and is considered the simplest low hydroxyl carboxylic acid and bulk chemical [1]. The application range of LA is very wide in areas such as food processing, the pharmaceutical industry, chemical industry, cosmetics and other industries [2]. Even in 2010, the U.S. Department of Energy issued a report that listed LA as a potential building block for the future [3]. There are two main production methods for LA, chemical synthesis, and biological fermentation [1,4,5]. Compared with chemical synthesis, biological fermentation can use low-cost biomass waste, such as kitchen waste [6], straw [7], and sludge [8] as substrates for microbial LA fermentation, which is more environmentally friendly, has low energy consumption, and can produce LA with higher optical purity [9,10]. Today, more than 90% of the world’s LA production comes from biological fermentation [11].

However, the production of LA by the biological fermentation method still faces many problems and challenges. In addition to the LA in the fermentation broth, there are also a large number of bacteria, proteins, residual sugars, inorganic salts and other impurities, and after the completion of fermentation, it is necessary to use reasonable extraction technology to separate the LA [12]. The extraction and purification of LA directly affects the quality and yield of the product. In addition, the cost of LA recovery and purification accounts for 50–80% of the total cost of LA production [13,14]. The purity required for food-grade is approximately 80–90% and more than 90% for the pharmaceutical grade, which considerably affects its price [15]. Therefore, the recovery of LA from fermentation broth is considered one of the biggest challenges in LA production and a bottleneck restricting LA production. As the composition of the LA fermentation broth is complex, the traditional LA separation process is subsequently complex, in which the acid and alkali consumption is large, the labor intensity is large, and many by-products are produced, which is not friendly to the environment [13]. Four different separation and purification methods, precipitation, solvent extraction, adsorption, and membrane separation, have been extensively investigated for LA recovery from fermentation broth [1]. As the most common and conventional method, the calcium LA crystallization-acid solution process still faces the problems of low lactate recovery efficiency (LRE) [16]. While the high cost and toxicity of the solvents towards microorganisms limit their use in in situ extraction processes [17,18]. Membrane separation technology has the advantages of high efficiency, no phase change, low energy consumption [19,20]. In addition, membranes can permit synchronous fermentation and purification in a single reactor. Membranes include microfiltration (MF), ultrafiltration, nanofiltration, reverse osmosis, and electrodialysis membranes. There is the problem that impurities in broth can quickly foul all membranes. However, the extent of fouling may be far less during microfiltration [21]. To meet the needs of LA industrial production, it is imperative to develop an efficient, low-pollution, low-energy lactic acid separation technology.

Another factor affecting the final cost of LA generated through fermentation technology is the price of raw materials, which can be as high as 40% of the LA cost [22,23]. Recently, the raised price of commercialized raw material has increased the cost of LA production, and affects the final product price [1]. Consequently, industries are turning their channels to more economic alternatives, one of which, kitchen waste, has been used to produce LA [24,25,26,27,28]. After the fermentation of kitchen waste, the broth is centrifuged, through which most of the particle suspension and bacteria in the broth are removed. However, there are still some suspended particulate matters in the broth, and the content of total sugar, protein and other substances is still high, which requires further processing. Until now, there has been little coverage and insufficient research about the purification of LA in the fermentation broth of kitchen waste adopting membrane separation technology. As the experience and reports of other garbage fermentation broths using MF membranes to purify LA indicated the effective performance of membranes, the use of an MF membrane to purify the LA in the fermentation broth of kitchen waste was carried out in this study.

This project used an MF membrane separation process system to separate and extract the LA in the fermentation broth of kitchen waste, and mainly studied the separation performance of the MF membrane on the LA fermentation broth; the turbidity removal efficiency (TRE), color removal efficiency (CRE), total sugar removal efficiency (SRE), protein removal efficiency (PRE) and LA recovery efficiency (LRE) were investigated. Meanwhile, the effect of operating pressure, fermentation broth pH and temperature on the filtration performance were investigated for optimizing the most suitable operating conditions for the filtration process. In addition, the cleaning effect of different cleaning modes, cleaning agents and cleaning time on the MF membrane was investigated, and the most suitable MF conditions were determined. Finally, the sustainability of stable MF performance under different cleaning cycles was explored.

## 2. Materials and Methods

### 2.1. LA Fermentation Liquid for Kitchen Waste

The LA fermentation broth used in the MF processes was derived from kitchen waste fermentation by *Montessori Enterococcus* bacteria. Kitchen waste was taken from the Hongboyuan Canteen of the University of Science and Technology, Beijing, located in Beijing, China, and the collected kitchen waste was first manually removed from the hard bones, plastic bags, paper towels and other impurities, and then put into the meat grinder to stir, grind, mix well and put into the self-sealing bag, and put into the −20 °C refrigerator for freezing [29]. Before fermentation, tap water was added to the kitchen waste in a 1:1 (*w*/*v*) ratio to adjust the concentration of total solids. The LA bacteria used in the fermentation process were *Montessori Enterococcus* CGMCC 22227, the inoculation ratio was 10%, the fermentation temperature was maintained at 43 °C, and the pH was adjusted to 6.8–7.0 with NaOH solution (10 mol/L) every 12 h.

After the fermentation was completed (84 h), the raw fermentation broth was centrifuged and separated by centrifuge (Hunan Hexi Instrument Equipment Co., Ltd., Changsha, China) (speed 12,000 rpm, centrifugation 10 min). After centrifugation, the bottom sedimentary substance was removed from the raw fermentation broth, and then the upper oily substance was removed through the separating funnel to obtain the fermentation broth required for the experiment. After centrifugation, the fermentation broth had the features of a pH of 6.2–6.3, an LA content of 63.96 g/L, a color of 1137 Hazen, a turbidity of 175 NTU, a protein content of 0.21 g/L, and a total sugar content of 3.33 g/L.

### 2.2. Experimental Apparatus

**Figure 1:**
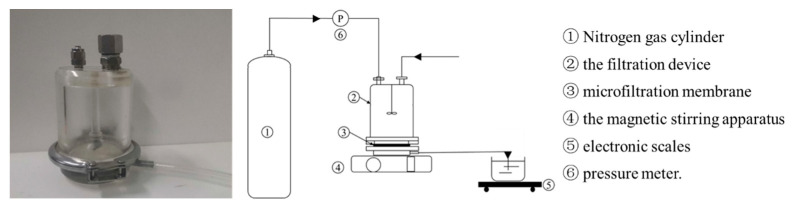
The illustration of the filtration configuration.

The MF device used in this study was a filtration cup (Shandong Bona Biotechnology Co., Ltd., Jinan, China), and the device is shown in Figure 1. The filtration cup adopted a flat membrane structure, and was equipped with a magnetic stirring rod to mix the liquid in the filtration cup, and high-pressure nitrogen (Beijing Huanyu Jinghui Gas Co., Ltd., Beijing, China) was used as the driving force to pressurize the system. Polymeric membranes are the most used in MF [30]. In this study, the MF membrane was made of polyvinylidene fluoride (PVDF), with a pore diameter of 0.1 μm, a diameter of 76 mm, and an effective filtration area of 45.36 cm^2^.

During the experiment, the MF membrane was loaded into the filtration cup, then, a certain amount of fermentation broth was loaded into the filtration cup, the magnetic stirrer below was turned on, and the speed was adjusted to 600 rpm. The nitrogen cylinder connected to the hose at the upper end of the filtration cup was opened, the pressure adjusted, and filtration performed until the outflow velocity of the liquid was less than 0.2 mL/min.

When the MF membrane was not in use, it was stored in a container filled with desiccant. After use, it was kept in a wet state in 0.5% formaldehyde solution. It was soaked in deionized water for 1 h before each use.

### 2.3. Analysis Indicators and Methods

#### 2.3.1. Turbidity, Chromaticity

The turbidity and color of the fermentation broth were measured using the water quality analyzer (Beijing Lianhua Yongxing Science and Technology Development Co., Ltd., Beijing, China).

The TRE and the CRE were calculated, as shown in Equation (1) and Equation (2).

TRE = (C_n0_ − C_n_)/C_n0_ × 100 (1)

where 

C_n_—the turbidity of the filtered transmissible fluid, NTU;

C_n0_—turbidity of the fermentation broth before filtration, NTU.

CRE = (C_h_ − C_h_)/C_h0_ × 100 (2)

where 

C_h_—the chromaticity of the filtered liquid, Hazen;

C_h0_—the chromaticity of the fermentation broth before filtration, Hazen.

#### 2.3.2. Determination of LA

The determination of LA in the fermentation broth was performed by high performance liquid chromatography (Shimadzu Corporation, Japan).

The calculation formula for the LRE of MF is shown in Equation (3).

LRE = (C_L_′V′)/(C_L0_V_0_) × 100 (3)

where 

C_L_′—the LA concentration of the fermented broth after filtration, g/L;

V′—product of fermented liquid after filtration, L;

C_L0_—LA concentration of fermentation broth before filtration, g/L;

V_0_—product of fermentation liquid before filtration, L.

#### 2.3.3. Determination of Proteins

Proteins in fermentation broths were determined using the Coomassie Brilliant Blue G-250 method [31].

The PRE was calculated, as shown in Equation (4).

PRE = (C_p0_ − C_p_)/C_p0_ × 100 (4)

where 

C_p_—the protein concentration in the filtered solution, g/L;

C_p0_—protein concentration in fermentation broth before filtration, g/L.

#### 2.3.4. Determination of Total Sugars

The determination of total sugars in the fermentation broth was carried out by the phenol-sulfuric acid method.

The SRE was calculated, as shown in Equation (5).

SRE = (C_so_ − C_s_)/C_so_ × 100 (5)

where 

C_s_—total sugar concentration in the filtered permeable solution, g/L;

C_s0_—total sugar concentration of fermentation broth before filtration, g/L.

#### 2.3.5. Determination of Membrane FLUX

Membrane flux was defined as the volume of liquid that permeates a membrane per unit area per time. The flux calculation formula is shown in Equation (6).

Flux(mL/min × m^2^) = V/(t × A) × 100 (6)

where 

V—through the liquid product, mL;

t—filtration time, min;

A—membrane area, m^2^.

#### 2.3.6. Membrane Cleaning Effect 

The membrane cleaning effect was expressed by the membrane pure water flux recovery efficiency (FRE), as shown in Equation (7).

FRE = (J_w_ − J_fw_)/(J_0_ − J_fw_) × 100 (7)

where 

J_0_— pure water permeation flux before membrane use, L/m^2^/h;

J_fw_—pure water permeation flux after membrane contamination, L/m^2^/h;

Jw—pure water permeation flux after membrane cleaning, L/m^2^/h.

## 3. Results and Discussion

### 3.1. The Effect of Operating Pressure on MF Performance

With a certain amount of fermentation broth injected into the filtration cup, the pressure was set to 60, 80, 100, 120 KPa, respectively, based on the pressure requirements (10–200 KPa) to carry out the MF separation [32]. Four sets of MF experiments were carried out to investigate the effect of different operating pressures on the removal performance of the MF membrane for turbidity, color, protein, and total sugar from fermentation broth, and LRE.

#### 3.1.1. The Change in MF Membrane Flux

**Figure 2:**
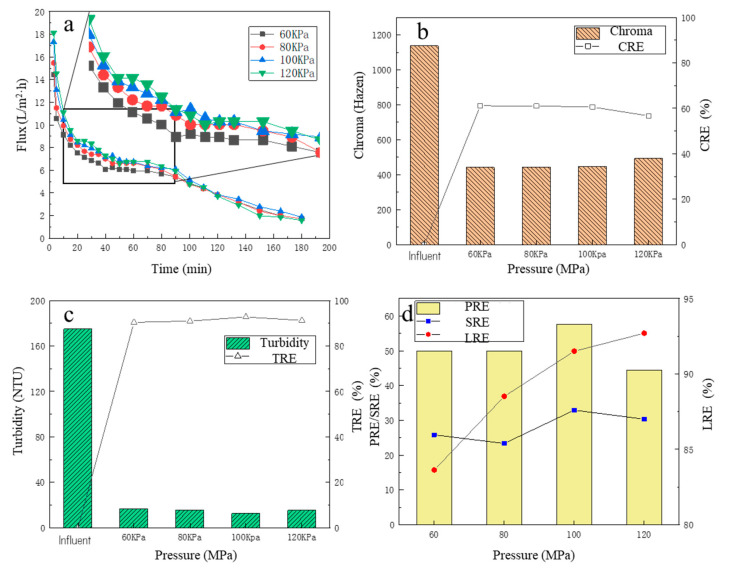
The effect of different pressures on filtration performance: (**a**) the flux change; (**b**) the chroma removal; (**c**) the turbidity removal; (**d**) the protein removal efficiency, the total sugar removal efficiency, and the lactic acid recovery efficiency.

Under different operating pressures, the change in membrane flux over time is shown in Figure 2a. There was a slight trend that the higher the operating pressure, the higher the membrane flux, especially for the initial period. For the first 10 min, it could be observed that the membrane flux decreased rapidly. This is because, in the initial period, some bacteria, colloids and macromolecular particles in the fermentation liquid were quickly adsorbed to the membrane surface, then the effective membrane holes were blocked, which was manifested by a rapid decline in membrane flux [33]. For the 10–40 min, it could be observed that although the membrane flux continued to decrease over time, the downward trend decreased significantly. For 40–90 min, the downward trend of MF membrane flux was further slowed down, and the membrane flux tended to be stable, which was due to the gradual formation of an adsorption layer on the membrane surface as the filtration proceeded [34,35]. After 90 min, the membrane flux began to decrease over time, which may be due to the high concentration of pollutants in the remaining fermentation broth, which further aggravated the membrane pollution and thus decreased the membrane flux. The average membrane fluxes under the four pressures (from high to low) were 6.68, 6.68, 6.41 and 5.94 L/(m^2^·h), respectively. It was observed that the average membrane flux generally increased with the increase in the operating pressure (from 60 KPa to 100 KPa), but when the pressure had increased to a certain extent (from 100 KPa to 120 KPa), the average membrane flux was almost stable. The reason may be that with the high operating pressure, the filter cake layer accumulated on the membrane surface was compacted, resulting in an increase in the thickness and density of the filter layer, which increased the filtration resistance and caused the membrane flux to increase insignificantly [32,35].

#### 3.1.2. The Removal Performance of Chroma and Turbidity

The color change in the fermentation broth is shown in Figure 2b, before and after the MF treatment. Before MF, the color of the fermentation broth was 1137 Hazen, and after the MF treatment, the color of the filtrate was significantly reduced, basically maintained between 440 and 480 Hazen, and the CRE was about 60%. With the increase in the operating pressure, the color of the filtrate showed an overall upward trend, but the change was minor. The minimum chromaticity was 441.7 Hazen at the operating pressure of 60 KPa. At the operating pressure of 120 KPa, the maximum chromaticity was 492.5 Hazen. Therefore, the MF treatment adopted in this study could effectively reduce the turbidity of the fermentation broth.

After centrifugation, most of the solid suspensions in the fermentation broth were removed, but many particle suspensions remained in the fermentation broth, and the turbidity of the fermentation liquid was still high (about 175NTU). Under different operating pressures, the turbidity of the filtrate and the TRE are shown in Figure 2c. After MF treatment, the turbidity of the filtrate decreased significantly, basically below 20 NTU, and the TRE reached more than 90%. It was obvious that the different operation pressures had a weak effect on the TRE, and at the operating pressure of 100 KPa, the filtrate turbidity was the lowest, only 12.61 NTU, and the TRE was 92.79%. At the operating pressure of 60 KPa, the filtrate turbidity was the highest, which was only 16.75 NTU, and the TRE reached 90.43%, which fully demonstrated that the MF operation could effectively reduce the turbidity of the fermentation broth.

#### 3.1.3. The Removal Performance of Protein and Total Sugar and Lactic Recovery Performance

Under four operating pressure conditions, the PRE and SRE are shown in Figure 2d. With the increase in pressure, the PRE increased. The PRE reached the highest value of 57.64% at 100 KPa, while the SRE had a fluctuating increase trend, and at 100 KPa, the SRE reached the maximum value of 32.93%. The reason why the PRE was higher than the SRE was partly because proteins, as hydrophobic compounds, are more readily adsorbed onto hydrophobic membrane surfaces than hydrophilic solutes due to hydrophobic interactions [36]. For the LRE, with the increase in pressure, the LRE increased significantly, and at 60 KPa, the LRE was only 83.63%, while at the pressure of 100 KPa, the LRE increased to 91.51%. This is because, with the increase in pressure, the share of filtrated flux also increased accordingly, resulting in clearer liquid, so its LA loss was lower. When the operating pressure continued to rise to 120 KPa, the LRE further increased to 92.70%, which was not a significant improvement, consistent with the law of change in membrane flux. Considering that the CRE, TRE, PRE and SRE of the fermentation broth were high under the operating pressure of 100 KPa with the relatively small energy consumption, the optimal operating pressure of the MF membrane was finally determined at 100 KPa.

### 3.2. The Effect of pH on MF Performance

In addition to the operating pressure, the pH of the fermentation broth also influences the MF performance. Four sets of MF experiments with the fermented broth placed in the filtration cup under different pHs (5, 6, 7, 8), adjusted by 6 mol/L HCl and 10 mol/L NaOH solution, and the nitrogen cylinder valve pressure of 100 KPa, were carried out. The turbidity, color, protein, total sugar, and LRE from the fermentation broth at different pHs were investigated.

#### 3.2.1. The Change in MF Membrane Flux

**Figure 3:**
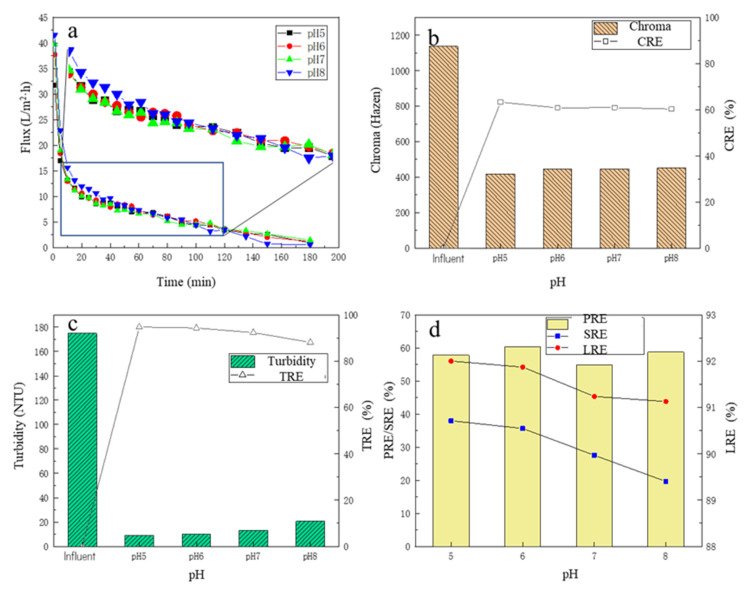
The effect of different pHs on filtration performance: (**a**) the flux change; (**b**) the chroma removal; (**c**) the turbidity removal; (**d**) the protein removal efficiency, the total sugar removal efficiency, and the lactic acid recovery efficiency.

Under different pH conditions, the changes in the membrane flux over time are shown in Figure 3a. Similar to Section 3.1.1, with the time proceeding, the membrane flux continued to decline, and the change trend in the membrane flux under the four pH conditions showed no obvious differences. There was a slight trend that the higher the operating pH, the higher the membrane flux, especially for the initial period. For the first 10 min, it could be observed that the membrane flux decreased rapidly. For 10–40 min, it could be observed that the membrane flux under the pH 8 condition was still the highest compared to the other three experimental sets, while the membrane flux under the pH 6 condition was slightly higher than those under the pH 5 and 7 conditions. After 40 min, the membrane flux at pH 8 decreased further and gradually decreased to less than that experienced under pHs of 5, 6, and 7. The reason may be that the pH affected the membrane–impurity interactions; it was reported that most polymers behave as cations in an acidic medium, and they have no interaction with positively charged compounds, whereas, in an alkaline medium, they have anionic behavior, having minimal interaction with negatively charged compounds [30,37,38]. Thus, in the initial stage, membranes could be used to separate compounds with the same charge based on the effect of electrostatic repulsion; then, the membrane underwent a period of slow fouling; in the later stage, the broth had higher impurity concentrations, which showed a stronger concentration gradient, which finally overcame the electrostatic repulsion, causing the rapid fouling of the membranes.

#### 3.2.2. The Removal Performance of Chroma and Turbidity

Under different pH conditions, before and after the MF treatment, the chroma change in the fermentation broth is shown in Figure 3b. With the increase in pH of the fermentation broth, the CRE in the MF process also showed a downward trend, and under the pH of 5, the CRE was the highest at 63.3%; the chroma of the fermentation broth after MF was only 417.27 Hazen. Under different pH conditions, the turbidity of the filtrate and the TRE are shown in Figure 3c. After the MF treatment, the turbidity of the fermentation broth decreased significantly, all below 20 NTU, and the TRE reached more than 92%. With the increase in the pH of the fermentation broth, the turbidity of the filtrate showed an overall upward trend, and the TRE showed a downward trend, but the overall change was small. When the pH was 5, the TRE was highest, reaching 94.8%, and the turbidity of the filtrate was 9.07 NTU. As stated in the Section 3.2.1, under higher pH conditions, due to electrostatic repulsion, some impurities would permeate through the membrane, and then enter the filtrate, which caused the impurities in the filtrate to increase.

#### 3.2.3. The Removal Performance of Protein and Total Sugar

Under four pH conditions, the SRE and PRE are shown in Figure 3d. With the increase in the pH of the fermentation broth, the SRE showed a gradual decreasing trend, when the pH of the fermentation broth was 5, the SRE was the highest, up to 37.99%, indicating that the MF membrane had a better removal effect on the total sugar under acidic conditions. There was no obvious law in the change in PRE, but overall, the change was small, and the highest PRE was 60.38% with the fermentation broth of pH 6. For the LRE, with the increase in pH, the LRE had a certain decrease, and when the pH = 5, the LRE was the highest, 92.00%. When the pH increased to 8, the LRE decreased to 91.13%. Overall, however, the change in LRE was small, no more than 1%. There were some reports that the membranes based on hydrophobic materials tend to interact easily with lipids, proteins, and peptides [38,39], which could partly explain why the PRE was higher than the SRE.

Considering various indicators, the chroma, turbidity, and SRE in the fermentation broth decreased with the increase in pH, and the LRE increased slightly with the decrease in pH, so the conditions with lower pH should be selected as much as possible. However, considering that the membrane flux decreases with the decrease in pH, and the adjustment of the pH of the fermentation broth requires the addition of hydrochloric acid, for the sake of cost, it should be as close as possible to the pH of the initial fermentation broth itself (6.2). Therefore, pH 6.0 was finally selected as the best pH condition in the MF process.

### 3.3. The Effect of Cleaning Method on Polluted Membrane Recovery

As can be seen from Figure 2a and Figure 3a, as the time passed by, there was a significant decrease in the membrane flux, which reflected the aggravation of the membrane blocking. In practice, to reduce the operating costs of the filtration process, the contaminated membrane should be cleaned to recover the membrane flux to some extent for prolonging the longevity of the membrane. Membrane cleaning methods can be divided into physical cleaning, chemical cleaning, biological cleaning and so on. Physical cleaning includes forward cleaning, reverse cleaning, ultrasonic cleaning, etc. Among these methods, hydraulic cleaning and gas–liquid cleaning technology are currently widely used as physical cleaning technologies.

#### 3.3.1. The Influence of Cleaning Mode on Cleaning Performance

In this study, four kinds of cleaning modes were conducted to investigate the membrane flushing performance, and they were: 3 min forward pressurized cleaning; 3 min reverse pressurized cleaning; 1.5 min forward pressurized cleaning + 1.5 min reverse pressurized cleaning; and 1.5 min reverse pressurized cleaning + 1.5 min forward pressurized washing.

The operation process was as follows: (1) first, a brand new MF membrane was taken and soaked in deionized water for 1h, and the J_0_ measured at 100 KPa; (2) the MF membrane was put into the filtration cup, 60mL of fermentation broth was added, and filtered at 100 KPa for 30 min; (3) the remaining fermentation liquid was poured out, the filtration cup was washed with deionized water, deionized water was added, the pressure adjusted to 100 KPa, and the J_fw_ measured; (4) the remaining liquid in the filtration cup was poured out, the filtration cup cleaned, deionized water added, adjusting the pressure to 100 KPa; then, it was forward pressure cleaned for 3 min and the J_w_ measured. The FRE was calculated according to the J_0_, the J_fw_, and the J_w_ based on Equation (7). The cleaning operations for the other three modes were the same as that for the forward pressure model.

**Figure 4:**
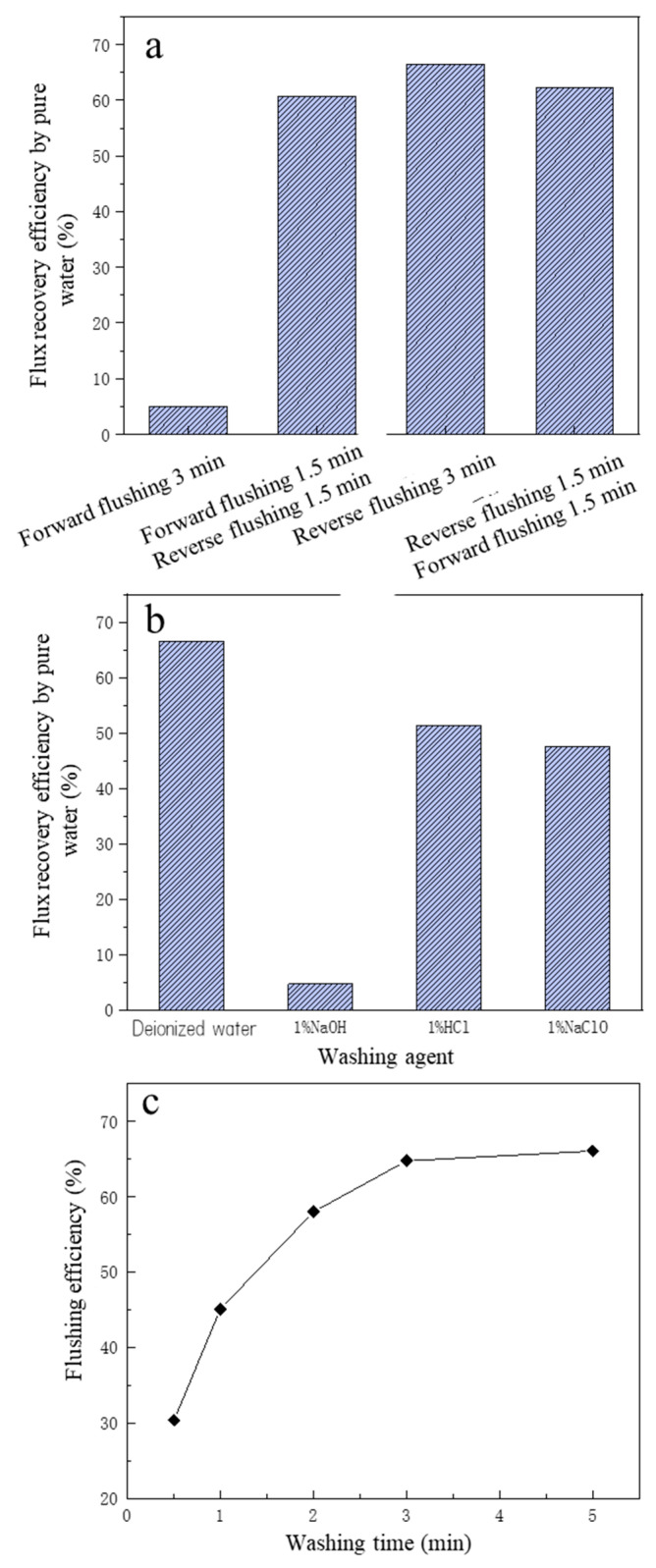
The effect of different flushing operations on flux recovery efficiency of microfiltration: (**a**) the flushing methods; (**b**) the flushing agents; (**c**) the effect of washing time on flushing efficiency.

The cleaning effect of the four cleaning methods on membrane MF pollution is shown in Figure 4a. The pure water FRE of forward pressurized cleaning was the worst, and the pure water FREs of forward pressurized cleaning for 1.5 min + reverse pressurized cleaning for 1.5 min mode and forward pressurized cleaning for 1.5 min + reverse pressurized cleaning for 1.5 min mode were close, but both were less effective than reverse pressurized cleaning for 3 min. The reason may be that most of the pollutants on the surface of the MF membrane were substances with large particle size, and their particle size was greater than the pore size (0.1 μm) of the MF membrane itself, with the result that in the forward pressurized cleaning mode, most of the polluting substances still stayed on the surface of the MF membrane, making the membrane pollution impossible to remove [40]. When reverse pressurized cleaning was carried out, most of the pollutants accumulated on the surface of the membrane were washed away by the water flow, and the longer the backwash time, the better the removal effect of membrane pollution. Therefore, backwashing was selected as the cleaning method of the MF membrane.

#### 3.3.2. The Influence of Cleaning Agent on Cleaning Performance

Chemical cleaning is generally required when backwashing is unable to remove the foulants or restore the membrane flux [36]. To further improve the FRE of the MF membrane, four cleaning agents were selected to clean the contaminated MF membrane. The four cleaning agents were deionized water, 1% HCl solution, 1% NaOH solution, 1% NaClO solution; the cleaning method was reverse pressurized cleaning, the pressure was 100 KPa, and the time was 3min. The removal performance of the four kinds of cleaning agents is shown in Figure 4b.

From Figure 4b, the reverse pressurized cleaning effect of deionized water was the best, and the FRE was 66.51%, indicating that for the MF membrane used in this study, compared with chemical cleaning, the effect of physical cleaning was better. The cleaning effect of 1% HCl was second, and the FRE was 51.39%, which may have been because some insoluble substances become soluble substances under acidic conditions, so that membrane pollution was reduced. The NaClO cleaning effect was third after HCl cleaning, and the FRE was 47.51%, which may be due to NaClO solution having strong oxidation properties, so that some organic pollutants were oxidized and decomposed [41]. The NaOH cleaning effect was the worst; the reason may be that the diaphragm reacted with NaOH, affecting the membrane pore structure and flux, based on relevant studies that showed that NaOH solution will react with PVDF membrane material, causing the diaphragm to appear brownish and discolored [42]. It was reported that HCI had a moderate effect and NaOH had the weakest effect on cleaning [36,43]. On the other hand, it may be because that under the effect of NaOH, certain calcium salts and lactate salts formed complexes, which combined with organic matter such as proteins to form aggregates. Then the formed aggregates were strongly absorbed by the membrane surface or membrane pores, making the membrane flux difficult to recover [44]. Compared with HCl, NaOH and NaClO, deionized water is cheaper, and the FRE was highest, so the deionized water pressurized backwash was finally selected as the cleaning method of the MF membrane.

#### 3.3.3. The Influence of Cleaning Time on Cleaning Performance

In addition to the cleaning method and cleaning agent, the cleaning time also has a greater impact on the cleaning effect. After determining the cleaning agent and cleaning method (deionized water backwash), to further improve the FRE, the influence of deionized water backwash time on the cleaning effect was investigated. As can be seen from Figure 4c, within the first 3 min of deionized water backwashing, the membrane FRE increased significantly with the increase in cleaning time. When the deionized water backwashing time was 30 s, the membrane cleaning efficiency was only 30.36%, and when the cleaning time reached 3 min, the membrane cleaning efficiency rose to 66.51%, which was about doubled. However, when the cleaning time exceeded 3 min, the membrane FRE basically no longer increased with time, and the cleaning efficiency was 67.64% even when the cleaning time was 5 min. Compared with the cleaning time of 3 min, the FRE just increased by 1.3%, and the increase was weak. Therefore, to save cleaning time and cleaning water, the more reasonable cleaning time for deionized water backwashing should be 3 min.

#### 3.3.4. MF Membrane Surface Morphology Analysis

**Figure 5:**
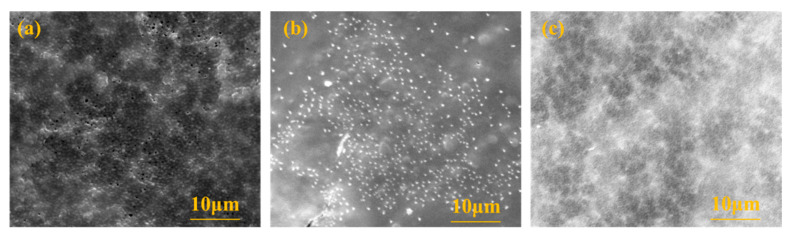
The surface observation of the microfiltration membrane by SEM instrument: (**a**) the new membrane; (**b**) after blocking; (**c**) after flushing with deionized water.

To study the morphology of the membrane surface before and after MF membrane cleaning, the membrane surface was determined by scanning electron microscopy, and the results are shown in Figure 5. As can be seen from the figure, some loose pores could be observed on the membrane surface of the new MF. After contamination, the membrane pores became invisible due to contaminants, and some particulate matter accumulated on the membrane surface. After backwashing with deionized water, the particles on the surface of the membrane were reduced, but a small number were still present. At the same time, the membrane pores were still blocked, indicating that deionized water could only remove part of the deposits on the surface of the membrane, and the contaminants blocked in the membrane pores could not be effectively removed, so the increase in the FRE of the membrane after only washing with water was not obvious.

### 3.4. The Effect of Cleaning Cycle on MF Membrane Performance

**Figure 6:**
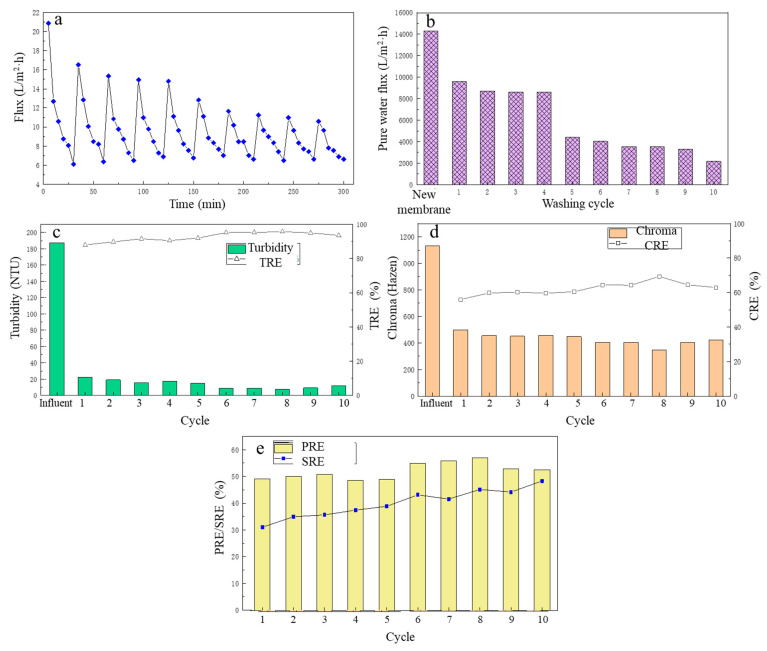
The effect of different flushing cycles on filtration performance: (**a**) the flux change; (**b**) the pure water flux; (**c**) the turbidity removal; (**d**) the chroma removal; (**e**) the protein removal efficiency and the total sugar removal efficiency.

After the MF membrane cleaning mode was determined, to further analyze the membrane flux change and treatment performance of the MF for fermentation broth after several cleaning phases, a 10-cycle MF experiment of the fermentation liquid was carried out, and each cycle was carried out for 30 min, recording the change in the membrane flux over time when the MF membrane filtered the lactate fermentation liquid in each cycle. After filtration was complete, the contaminated MF membrane was backwashed with deionized water and the pure water flux of the MF membrane was recorded after each cleaning. The treatment performance during each MF cycle was analyzed. The specific results are shown in Figure 6.

From Figure 6a, after the first cleaning, the flux of the LA fermentation broth filtered by the MF membrane in the second cycle decreased to a certain extent compared with the first cycle. In the next three MF cycles (that is, from cycle 3 to cycle 5), although the MF membrane flux continued to decline, the average membrane fluxes of cycles 3–5 were 9.74 L/(m^2^·h), 9.72 L/(m^2^·h), 9.68 L/(m^2^·h), respectively. However, in general, the MF membrane flux did not change much in the initial period, and it was basically maintained at a relatively high level, which fully explained the stability of the deionized water backwash effect. However, from the sixth cycle, the MF membrane flux began to decrease at a faster speed, and in the ninth cycle, the average membrane flux was the lowest, only 8.18 L/(m^2^·h). The same was true for the change in the pure water flux of the MF membrane, as shown in Figure 6b. After the first cleaning, the pure water flux had a large decrease, and then until the fourth cleaning, the pure water flux basically did not change much. Similarly, after the fifth cleaning, the pure water flux of the MF membrane had decreased significantly, and continued to decline, and the pure water flux of the MF membrane after 10 cleaning phases was only 2169.6 L/(m^2^·h). This is because the deionized water backwashing could only remove suspended solids deposited on the surface of the membrane. For the first five filtration cycles, the main cause of membrane pollution was the pollution layer accumulated on the membrane surface, and after water backwashing, the contaminated layer was effectively removed, and the membrane flux was effectively restored. However, with the increase in filtration cycles and time, the pollution inside the membrane pores gradually accumulated and was difficult to remove with the water backwash, resulting in a significant decrease in membrane flux.

Over the 10 filtration cycles, the removal of turbidity and chroma of the fermentation broth is shown in Figure 6c and Figure 6d, respectively. In general, the TRE and CRE of the fermentation broth by the MF membrane increased with the increase in number of filtration cycles. During these cycles, the TRE was maintained between 88 and 96%, and the CRE was maintained between 56 and 70%. During the eighth filtration cycle, TRE and CRE reached their highest, at 95.78 and 69.35%, respectively.

Over 10 filtration cycles, the PRE and SRE from the fermentation broth by the MF membrane are shown in Figure 6e. In the first five filtration cycles, the PRE change was small and basically maintained at about 50%, and in the last five MF cycles, the PRE first increased with the increase in the filtration cycle, reaching a maximum 57.02 % in the eighth MF cycle. Then over the next two cycles, it gradually decreased, but still was around 52%. The SRE showed an overall upward trend with the increase in number of the filtration cycles, with the SRE of 31.11% in the first MF cycle. As the number of filtration cycles increased, it was in the 10th MF cycle that the SRE reached its highest at 48.39%. The reason for the analysis results may be that with the increase in the number of filtrations, the membrane pollution inside the MF membrane gradually accumulated, which reduced the size of the membrane pores and increased the retention rate of the membrane, thereby increasing the SRE.

### 3.5. The Whole Evaluation of the MF in This Study

**Figure 7:**
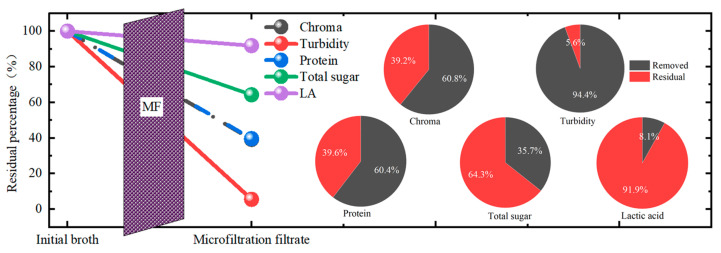
The whole removal performance of the microfiltration membrane separation and the removal percentage of each component.

The whole removal performance of the MF in this study was summarized, as shown in Figure 7. Among all the impurities, the MF membrane achieved the best removal performance for turbidity with a TRE of more than 94%, followed by the chroma and protein, with a removal efficiency of more than 60%. The total sugar removal performance was the worst. From these, it is obvious that for the purification of the LA in kitchen waste fermentation broth, the MF process could achieve the effect to some extent, while it was not enough to realize higher purity. The combination of MF with other post-treatment processes was needed. In addition, after the microfiltration, the LA loss was about 8.1%. Thus, in future research, the effort to lower the loss of LA during filtration should be considered.

## 4. Conclusions

In this study, for the purification of kitchen waste fermentation broth, the optimum operation parameters were determined as a pressure of 100 KPa, pH of 6.0, and a flushing mode of backwashing with deionized water for 3 min. The best performance was achieved with the CRE, TRE, PRE and SRE of 60, 92.8, 57.64 and 32.93%, respectively. This study paves the way for the purification of kitchen waste fermentation broth with MF membrane technology. The obtained results have meaning as a reference for future research. In all, the MF process is promising as an effective separation method for the preliminary purification of kitchen waste fermentation broth, and this study supplies the basic reference for further exploration.

## Data Availability

A statement was submitted.
